# A laboratory-based selected logistic model for early prediction of 28-day mortality in patients with septic shock

**DOI:** 10.3389/fmed.2026.1864043

**Published:** 2026-10-13

**Authors:** Liying Zhang, Pu Chen, Jie Li, Weilei Xiao, Wentao Zhou

**Affiliations:** 1 The Blood Transfusion Department, the First People’s Hospital of Yibin City, Yibin, China; 2 The Laboratory Department, the Third People’s Hospital of Yibin City, Yibin, China; 3 The Intensive Care Unit, the First People’s Hospital of Yibin City, Yibin, China; 4 The Laboratory Department, the First People’s Hospital of Yibin City, Yibin, China; 5 School of Laboratory Medicine, North Sichuan Medical College, Nanchong, China

**Keywords:** bootstrap validation, D-dimer, lactate, nomogram, PaO2/FiO2, prediction model, sepsis, septic shock

## Abstract

**Background:**

Early risk stratification in septic shock requires transparent models based on variables available soon after clinical diagnosis of septic shock. We reanalysed a retrospective cohort using the original screening strategy: candidate predictors with univariable *P* < 0.05 were entered into multivariable logistic regression, and variables with multivariable *P* < 0.05 were retained in the final model.

**Methods:**

The analysis included 250 patients with complete 28-day outcome and laboratory data. The outcome was 28-day mortality. Candidate predictors comprised demographics, comorbidities, complete blood count, chemistry, coagulation, inflammatory markers, arterial pH, and PaO2/FiO2. Laboratory values were treated as the first available values within 24 h after clinical diagnosis of septic shock. The final fixed model and the complete model-development pipeline were internally validated using 1,000 bootstrap resamples. Discrimination, calibration, Brier score, ROC-derived cutoff, decision curve analysis, LAR comparison, restricted cubic spline analysis, VIF, and LASSO sensitivity analysis were reported.

**Results:**

Among 250 patients, 73 died by day 28 (29.2%). Univariable screening identified 12 candidate predictors, and multivariable screening retained lactate, D-dimer, and PaO2/FiO2. The final equation was logit(*P*) = 0.412145 + 0.250015 × Lactate + 0.046852 × D-dimer - 0.016820 × PaO2/FiO2. The apparent AUC was 0.902 (95% CI 0.864–0.940), and the optimism-corrected AUC for the fixed final model was 0.897. When the complete model-development pipeline was repeated within 1,000 bootstrap resamples, the optimism-corrected AUC was 0.875. The final model had higher discrimination than LAR alone [0.902 (0.864–0.940) vs. 0.719 (0.645–0.793); DeLong *P* < 0.001].

**Conclusions:**

A selected logistic model using lactate, D-dimer, and PaO2/FiO2 provided internally validated early risk estimates for 28-day mortality. External validation is required before routine clinical use.

## Introduction

Sepsis remains a leading contributor to global mortality and health-care burden, and septic shock represents a particularly high-risk subgroup (1–4). The Sepsis-3 framework defines septic shock using vasopressor requirement and elevated lactate after adequate fluid resuscitation, but retrospective clinical datasets may not always capture all elements of this construct with sufficient precision (2, 3). For prognostic model development, transparent case ascertainment is essential because the way the cohort is defined can directly affect predictor distributions, model coefficients, and transportability.

Existing bedside severity scores such as qSOFA, SOFA, APACHE II, and NEWS2 are useful clinical frameworks, but they require physiologic, neurologic, treatment, or organ-dysfunction variables that may not be uniformly available in retrospective laboratory datasets (5–8). In contrast, routine laboratory and arterial blood gas variables are frequently measured early during shock care and may provide an objective, reproducible basis for early risk estimation. Lactate reflects metabolic stress and impaired perfusion (9–11), D-dimer reflects coagulation and fibrinolytic activation in sepsis-associated endothelial and microvascular injury (12–15), and PaO2/FiO2 captures oxygenation impairment and respiratory organ dysfunction (16, 17).

Several studies have proposed the lactate-to-albumin ratio (LAR) as a simple prognostic marker in sepsis and septic shock (18–22). LAR is clinically attractive because it combines a perfusion/metabolic marker with a negative acute-phase and nutritional marker, but it does not directly represent coagulation or oxygenation domains. Therefore, comparison with a selected multivariable model is important. We reanalysed the cohort using the original variable-screening strategy, then evaluated discrimination, calibration, bootstrap optimism, decision-curve net benefit, LAR comparison, restricted cubic spline nonlinearity, variance inflation factors, and LASSO sensitivity analysis. Reporting follows TRIPOD + AI 2024 principles, with attention to risk-of-bias concerns relevant to prediction model studies (23, 24).

## Methods

### Study design and setting

This was a retrospective single-center observational prediction-model study at the First People's Hospital of Yibin City, which enrolled adult patients hospitalized between January 2020 and November 2025. The study was approved by the Institutional Ethics Committee of the First People's Hospital of Yibin City (approval number: 2026(81)), and the requirement for individual informed consent was waived by the ethics committee for this retrospective chart-review analysis. The manuscript was prepared in accordance with the TRIPOD + AI reporting guideline for clinical prediction models (23). No prospective pre-planned specimen collection or research-specific additional laboratory testing was performed in this study. All laboratory and arterial blood gas measurements were generated as part of routine clinical patient care. Only de-identified historical electronic medical record data were retrospectively extracted for analysis after ethical approval was obtained.

Before the current minor revision analyses, the dataset was locked after author-led verification of the updated source dataset. All statistical analyses reported in the revised manuscript were regenerated from this single locked analytic dataset.

### Participants

Eligible patients were adults with septic-shock diagnosis documented in the medical record. For this retrospective study, chart-documented septic shock was operationally confirmed from routine-care records by the presence of suspected or confirmed infection together with infection-associated circulatory failure requiring vasopressor support after initial resuscitation or during ongoing shock management. Eligibility was determined based on the treating clinicians’ chart diagnosis and subsequent record review. Duplicate hospital admissions were excluded, and only the first clinical encounter was retained for patients with multiple admissions. Patients were required to have complete 28-day outcome information as well as at least one laboratory and arterial blood gas measurement obtained within 24 h following the clinical diagnosis of septic shock. If multiple test results existed within this predefined 24-hour window, the earliest measurement recorded after diagnosis was used for analysis. The 11 patients excluded for not meeting septic shock eligibility criteria were those whose records did not confirm infection-associated shock requiring vasopressor support on review, including records with noninfectious shock attribution, transient hypotension without vasopressor-supported septic shock, or insufficient documentation of septic shock.

### Predictors and outcome

Candidate predictors were age, sex, hypertension, diabetes mellitus, WBC, PLT, HGB, lactate, creatinine, albumin, PT, INR, D-dimer, CRP, PCT, pH, and PaO2/FiO2. Predictors were selected because they were routinely available within early care and represented demographic, comorbidity, hematologic, metabolic, renal, coagulation, inflammatory, acid-base, and oxygenation domains. In the source file, PaO2/FiO2 was computed as PO2 divided by FiO2 expressed as a fraction. PCT was treated as ng/mL. Hypertension was defined as a documented pre-existing diagnosis of hypertension in the electronic medical record or long-term use of antihypertensive medication before admission. Elevated blood pressure recorded only during the acute hospitalization was not used to newly define hypertension. Diabetes mellitus was defined as a documented pre-existing diagnosis of diabetes mellitus in the electronic medical record or pre-admission use of insulin or oral hypoglycemic agents. Stress hyperglycemia during the acute septic episode alone was not used to define diabetes mellitus. The outcome was 28-day all-cause mortality.

### Fluid resuscitation

Initial resuscitation was performed according to local clinical practice broadly consistent with contemporary sepsis guidelines. Crystalloids were used as the first-line resuscitation fluid. For patients with sepsis-induced hypoperfusion or septic shock, early crystalloid resuscitation was recommended, generally targeting approximately 30 mL/kg within the first 3 h when not contraindicated. The actual volume and rate of fluid administration were individualized according to clinical assessment, cardiac and renal function, pulmonary edema risk, and hemodynamic response. Vasopressors were initiated when hypotension persisted despite initial fluid resuscitation, with norepinephrine as the preferred first-line agent when clinically appropriate. Detailed treatment-process variables, including fluid balance, exact timing of resuscitation relative to laboratory sampling, albumin administration, blood product transfusion, and ventilator settings, were outside the prespecified laboratory-based predictor set.

### Missing data and sample size

A total of 287 potentially eligible patients were screened, and 250 patients were included in the final analytic cohort after exclusion of 37 patients. Exclusions were due to not meeting septic shock eligibility criteria (*n* = 11), missing laboratory or arterial blood gas data within 24 h after clinical diagnosis of septic shock (*n* = 13), missing or uncertain 28-day outcome (*n* = 8), and duplicate admission or other prespecified exclusion (*n* = 5). The supplied analytic dataset contained no missing values in required variables. Complete-case analysis was therefore used, but complete-case cohort construction may introduce selection bias. The sample size was obtained through retrospective consecutive enrolment of eligible patients. Although the final refitted model contained three predictors and 73 mortality events, the development process evaluated 17 candidate predictors and entered 12 variables into multivariable screening. Therefore, the sample-size limitation and potential selection-related overfitting should be interpreted in relation to the full development process rather than only the final three-variable model. No formal pre-study sample-size calculation was performed.

### Model development and validation

Following the original modeling strategy, each candidate predictor was first evaluated using univariable logistic regression. Variables with univariable *P* < 0.05 were entered simultaneously into multivariable logistic regression. Variables with multivariable *P* < 0.05 were retained and refitted as the final model. Odds ratios were reported per one-unit increase on the native measurement scale. The final model equation included the intercept and beta coefficients. Because *P*-value screening may be unstable in modest datasets, LASSO logistic regression was prespecified as a sensitivity analysis rather than as a replacement model (25, 26). To address optimism related to predictor selection, an additional full-pipeline bootstrap repeated the complete development workflow in each bootstrap resample, including univariable screening, multivariable *P*-value selection, final refitting, and performance estimation in the bootstrap and original samples.

Model performance was assessed using AUC with 95% CI, Brier score, calibration intercept, calibration slope, and calibration plots. Internal validation used 1,000 bootstrap resamples to estimate optimism in discrimination and overall accuracy, following standard prediction-model validation principles (25, 26). Fixed-final-model bootstrap validation was reported for the final three-variable model, and full-pipeline bootstrap validation was reported as a supplementary analysis of the complete model-development procedure. A Youden optimal predicted-probability cutoff was reported with sensitivity, specificity, PPV, NPV, accuracy, and Youden index. Clinical utility was assessed using decision curve analysis over threshold probabilities from 0.01 to 0.80 (27). LAR was defined as lactate/albumin and compared with lactate alone, albumin alone, lactate plus albumin, and the final model. Restricted cubic splines assessed nonlinearity in final predictors. VIF assessed multicollinearity. All statistical analyses were performed using R version 4.5.1 (R Foundation for Statistical Computing, Vienna, Austria). The principal packages included pROC for ROC analysis, rms for logistic-model validation and restricted cubic splines, glmnet for LASSO regression, and car for variance inflation factors; bootstrap procedures were implemented with reproducible R scripts using 1,000 resamples.

## Results

### Baseline characteristics

Of 287 screened patients, 37 were excluded and 250 were included in the final analytic cohort (Figure 1). The cohort included 177 survivors and 73 non-survivors at 28 days, corresponding to a 28-day mortality of 29.2%. Overall, age was 67.00 [53.25–76.00], and 137 (54.8%) were male. Median lactate was 4.05 [2.60–6.40], D-dimer was 6.25 [2.40–16.28], and PaO2/FiO2 was 220.85 [157.18–287.50]. Baseline characteristics by survival status are shown in Table 1.

**Figure 1:**
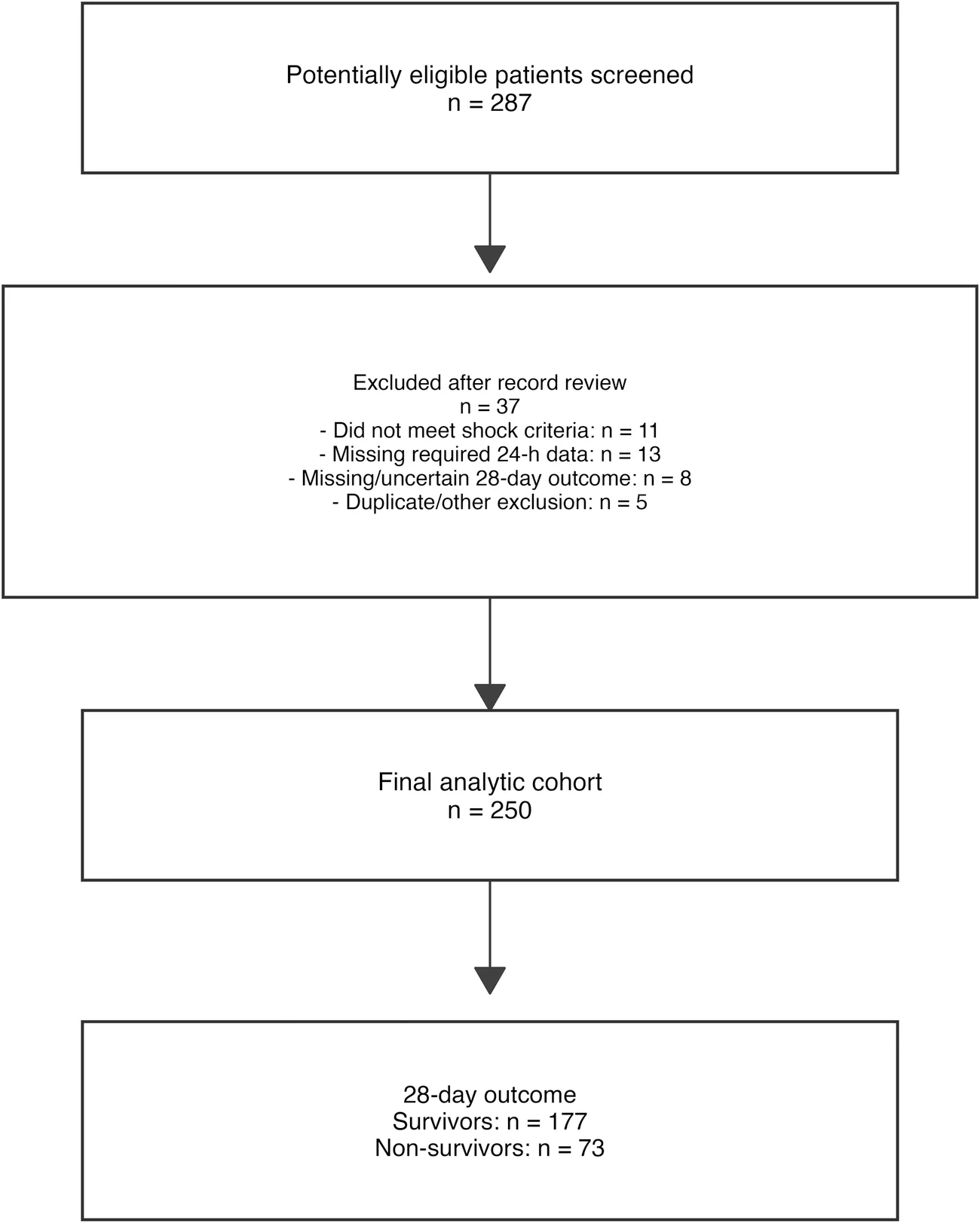
Patient screening and selection flow diagram.

**Table 1:** Baseline characteristics by 28-day survival status.

Variable	Overall	Survivor	Non-survivor	*P* value
Number of patients, n	250	177	73	
Sex, male	137 (54.8%)	91 (51.4%)	46 (63.0%)	0.094
Hypertension	88 (35.2%)	52 (29.4%)	36 (49.3%)	0.003
Diabetes mellitus	76 (30.4%)	56 (31.6%)	20 (27.4%)	0.507
Age, years	67.00 [53.25–76.00]	62.00 [50.00–74.00]	74.00 [61.00–82.00]	<0.001
WBC, ×10^9/L	14.98 [9.94–20.87]	15.67 [10.92–21.49]	12.16 [5.11–20.24]	0.007
PLT, ×10^9/L	132.50 [70.25–201.75]	142.00 [99.00–206.00]	89.00 [40.00–187.00]	0.001
HGB, g/L	107.37 ± 26.79	110.50 ± 25.95	99.79 ± 27.48	0.005
Lactate, mmol/L	4.05 [2.60–6.40]	3.60 [2.50–5.60]	5.90 [3.80–9.70]	<0.001
Creatinine, μmol/L	130.25 [88.80–242.87]	123.10 [83.30–200.00]	182.30 [107.00–296.00]	0.003
Albumin, g/L	30.45 [27.30–34.53]	31.40 [28.00–35.10]	28.50 [26.00–33.30]	0.004
PT, s	15.80 [14.10–17.98]	15.40 [13.90–17.40]	17.10 [15.00–21.40]	<0.001
INR	1.30 [1.14–1.50]	1.26 [1.12–1.44]	1.44 [1.24–1.83]	<0.001
D-dimer, mg/L	6.25 [2.40–16.28]	4.50 [2.10–9.00]	16.20 [6.30–30.50]	<0.001
CRP, mg/L	153.20 [88.82–247.23]	154.10 [83.50–258.70]	150.60 [107.80–227.00]	0.770
PCT, ng/mL	30.63 [4.15–104.93]	30.40 [6.14–106.20]	35.20 [1.16–80.26]	0.653
pH	7.43 [7.35–7.48]	7.44 [7.37–7.49]	7.36 [7.22–7.46]	<0.001
PaO2/FiO2, mmHg	220.85 [157.18–287.50]	251.11 [192.45–319.51]	147.17 [98.20–194.34]	<0.001

Continuous variables are presented as mean ± SD or median [IQR] according to distribution. Categorical variables are presented as *n* (%).

### Variable selection and final model

Univariable screening identified age, hypertension, PLT, HGB, lactate, creatinine, albumin, PT, INR, D-dimer, pH, and PaO2/FiO2 as candidates for multivariable modeling. In the multivariable model containing these screened variables, lactate, D-dimer, and PaO2/FiO2 remained statistically significant and were retained. The final formula was logit(*P*) = 0.412145 + 0.250015 × Lactate + 0.046852 × D-dimer - 0.016820 × PaO2/FiO2. The nomogram visualization of the final prediction model is presented in Figure 2. The complete univariable and multivariable logistic regression screening process is shown in Table 2. Variables with univariable *P* < 0.05 were entered into multivariable logistic regression, and lactate, D-dimer, and PaO2/FiO2 were retained because their multivariable *P* values were < 0.05. No substantial multicollinearity was detected, with all final-model VIF values below 5 (Supplementary Table S1).

**Figure 2:**
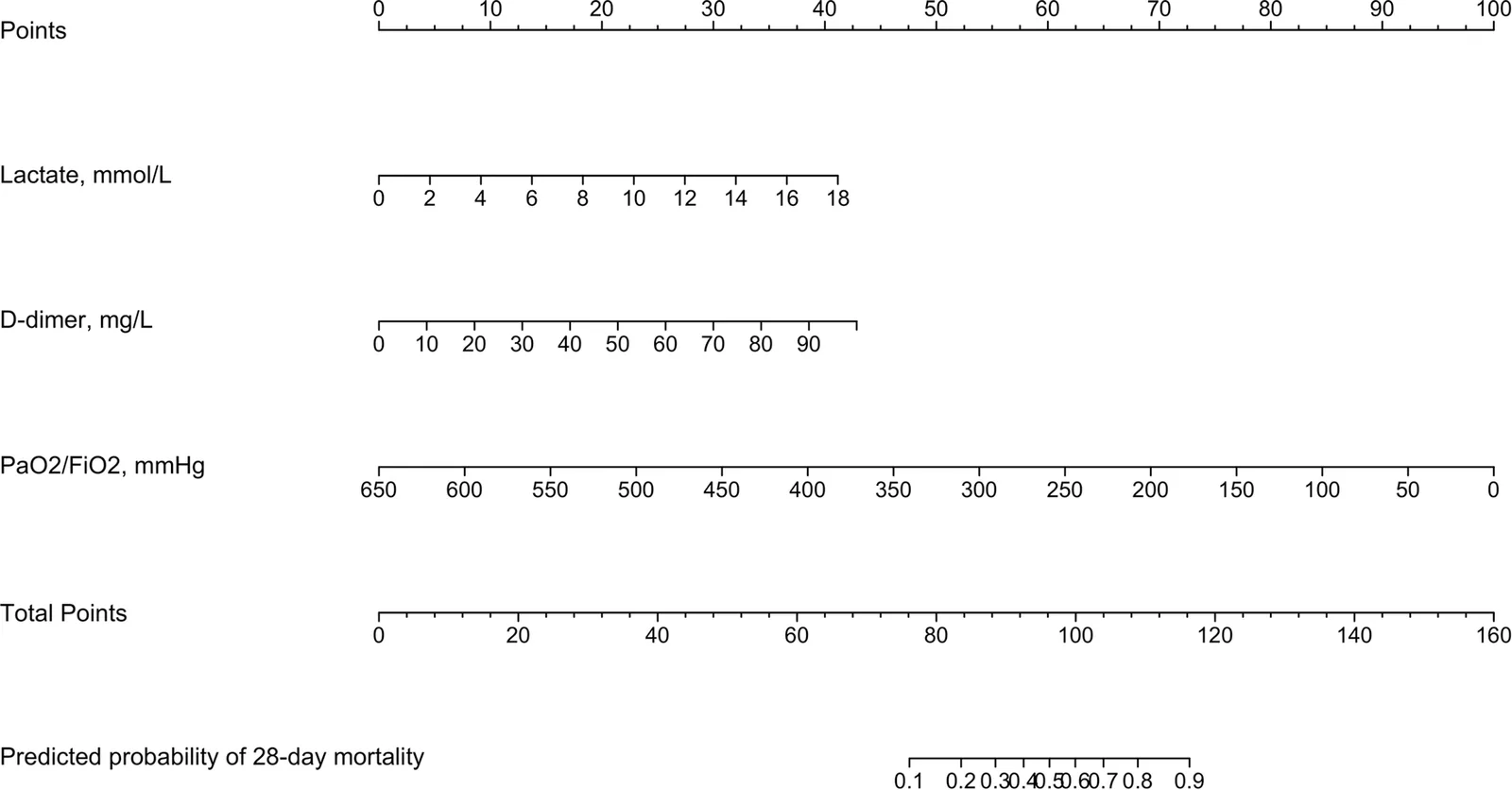
Nomogram for the final selected logistic regression model using lactate, D-dimer, and PaO2/FiO2.

**Table 2:** Univariable and multivariable logistic regression for 28-day mortality.

Variable	Univariable β	Univariable OR (95% CI)	Univariable *P* value	Multivariable β	Multivariable OR (95% CI)	Multivariable *P* value	Retained in final model
Age, years	0.0391	1.040 (1.020–1.060)	<0.001	0.0246	1.025 (0.997–1.054)	0.081	No
Sex, male	0.4763	1.610 (0.920–2.816)	0.095	—	—	—	No
Hypertension	0.8497	2.339 (1.334–4.100)	0.003	0.4429	1.557 (0.674–3.597)	0.300	No
Diabetes mellitus	−0.2041	0.815 (0.446–1.492)	0.508	—	—	—	No
WBC, ×10^9/L	−0.0149	0.985 (0.959–1.012)	0.268	—	—	—	No
PLT, ×10^9/L	−0.0038	0.996 (0.993–0.999)	0.015	−2e−04	1.000 (0.996–1.004)	0.912	No
HGB, g/L	−0.0157	0.984 (0.974–0.995)	0.005	−0.0135	0.987 (0.971–1.002)	0.095	No
Lactate, mmol/L	0.2575	1.294 (1.178–1.421)	<0.001	0.1349	1.144 (1.006–1.302)	0.041	Yes
Creatinine, μmol/L	0.0027	1.003 (1.001–1.005)	0.005	5e−04	1.001 (0.998–1.003)	0.696	No
Albumin, g/L	−0.0665	0.936 (0.890–0.984)	0.010	−0.0371	0.964 (0.887–1.046)	0.378	No
PT, s	0.1331	1.142 (1.063–1.228)	<0.001	−0.2578	0.773 (0.400–1.492)	0.443	No
INR	1.4918	4.445 (2.008–9.843)	<0.001	3.5902	36.241 (0.023–58222.999)	0.340	No
D-dimer, mg/L	0.0673	1.070 (1.045–1.095)	<0.001	0.0401	1.041 (1.011–1.072)	0.008	Yes
CRP, mg/L	0.0002	1.000 (0.998–1.003)	0.851	—	—	—	No
PCT, ng/mL	0.0018	1.002 (0.997–1.006)	0.434	—	—	—	No
pH	−5.0505	0.006 (0.001–0.060)	<0.001	−2.7779	0.062 (0.003–1.372)	0.078	No
PaO2/FiO2, mmHg	−0.0175	0.983 (0.978–0.988)	<0.001	−0.0175	0.983 (0.977–0.989)	<0.001	Yes

Variables with univariable *P* < 0.05 were entered into multivariable logistic regression. Variables with multivariable *P* < 0.05 were retained and refitted as the final model. ORs are shown per one-unit increase on the native measurement scale.

### Internal validation, ROC cutoff, and calibration

Bootstrap internal validation of the fixed final model showed apparent AUC 0.902 (95% CI 0.864–0.940), optimism-corrected AUC 0.897, apparent Brier score 0.114, optimism-corrected Brier score 0.119, and bootstrap optimism-corrected calibration slope 0.956 (Table 3). When the complete model-development pipeline was repeated in 1,000 bootstrap resamples, the optimism-corrected AUC was 0.875, the optimism-corrected Brier score was 0.132, and the optimism-corrected calibration slope was 0.809 (Supplementary Table S5). The Youden optimal predicted-probability cutoff was 0.361, with sensitivity 0.795, specificity 0.859, PPV 0.699, NPV 0.910, accuracy 0.84, and Youden index 0.653 (Supplementary Table S4; Figure 3). Calibration is shown in Figure 4.

**Table 3:** Bootstrap internal validation of the final model.

Metric	Estimate
Apparent AUC	0.902
Apparent AUC 95% CI	0.864–0.940
Optimism-corrected AUC	0.897
Apparent Brier score	0.114
Optimism-corrected Brier score	0.119
Apparent calibration intercept	0.000
Apparent calibration slope	1.000
Bootstrap optimism-corrected calibration slope	0.956
Somers’ Dxy, apparent	0.803
Bootstrap replicates completed	1000

Optimism was estimated using 1,000 bootstrap resamples for the fixed final model. A supplementary full-pipeline bootstrap repeated the complete variable-selection workflow in each resample.

**Figure 3:**
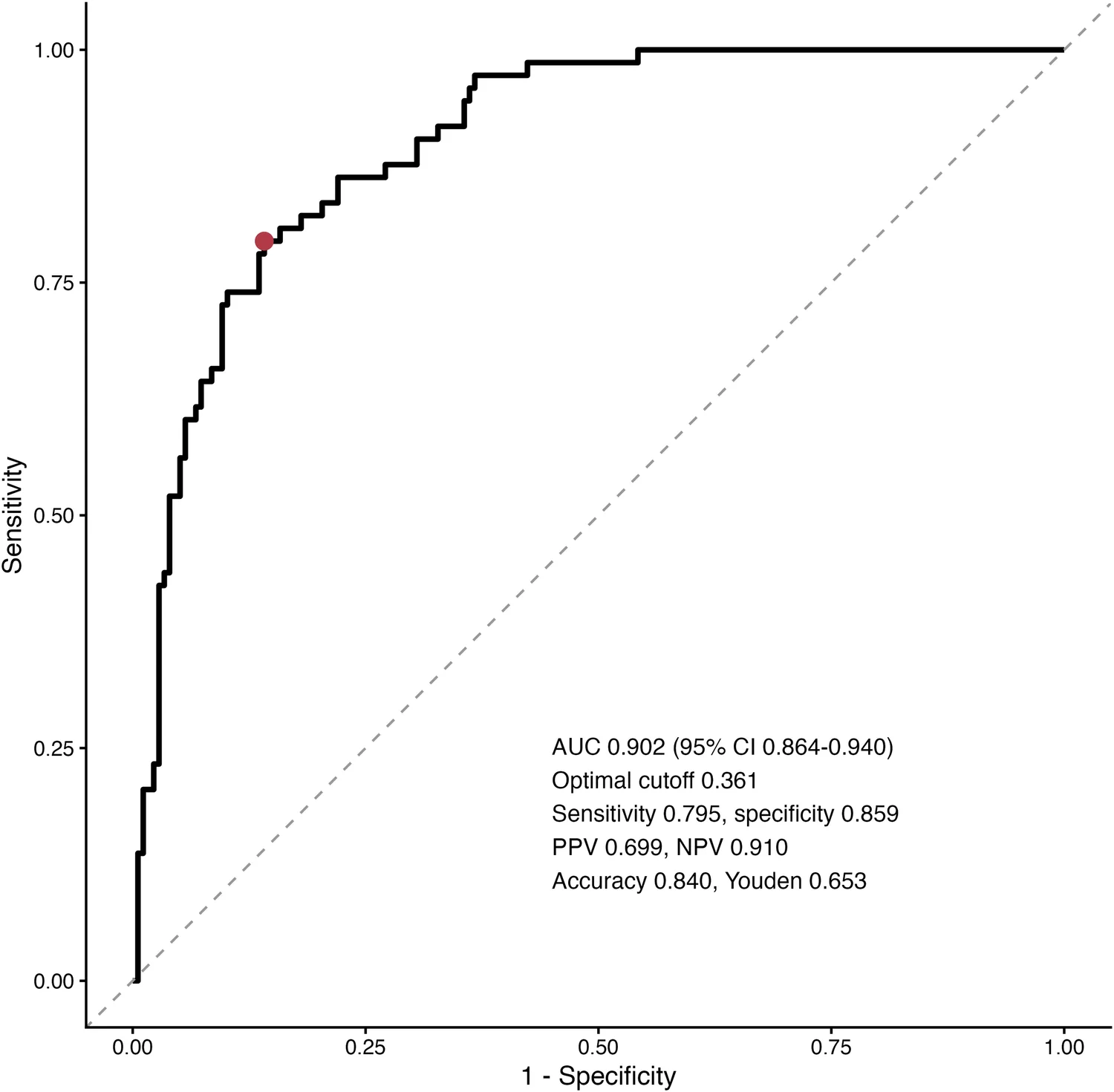
ROC curve for the final selected model. The marked point indicates the Youden optimal predicted-probability cutoff.

**Figure 4:**
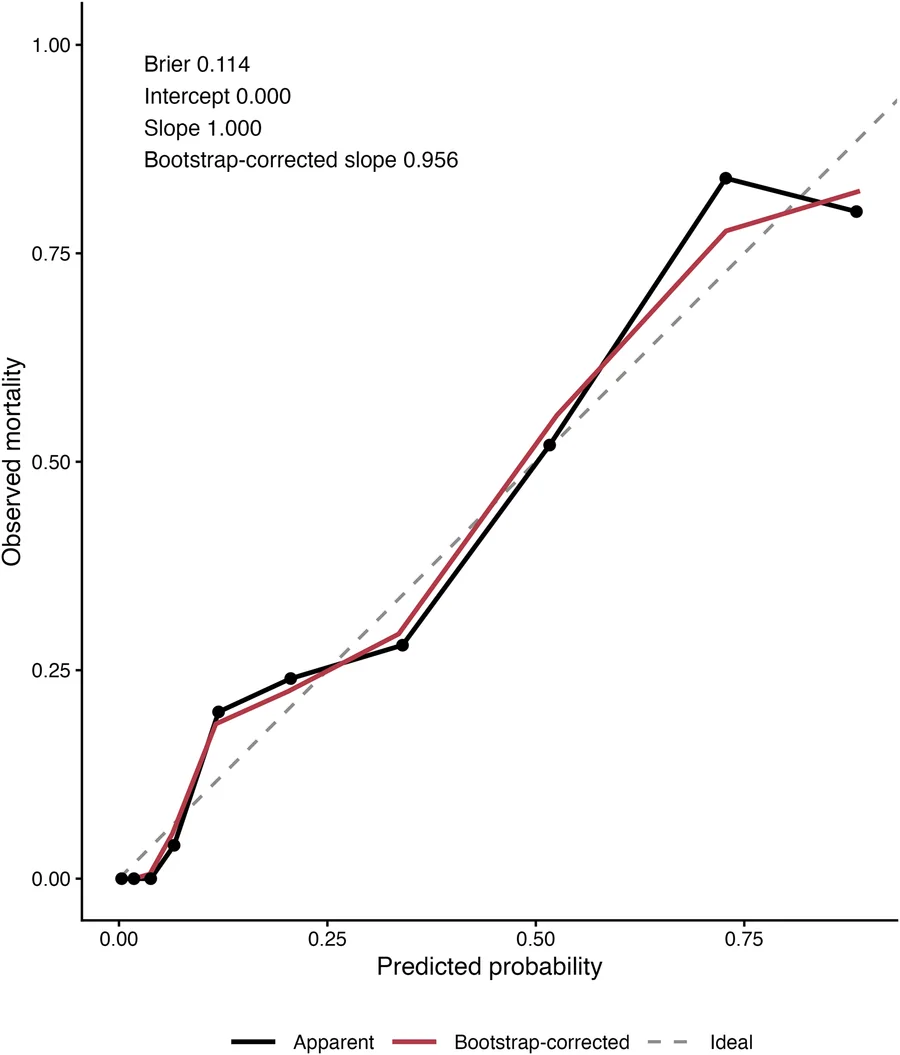
Apparent and bootstrap-corrected calibration curves for the final selected model. Calibration was internally validated using 1,000 bootstrap resamples.

### Comparison With LAR and sensitivity analyses

LAR alone had AUC 0.719 (0.645–0.793), whereas the final selected model had AUC 0.902 (0.864–0.940). The final model performed better than LAR alone by DeLong testing (*P* < 0.001; Table 4; Supplementary Figure S1). LAR was not significantly better than lactate alone in this dataset (DeLong *P* = 0.205).

**Table 4:** Comparison between LAR and the final selected model.

Model	AUC (95% CI)	Brier score	AIC	BIC	DeLong comparison	DeLong *P* value
Lactate alone	0.705 (0.631–0.780)	0.174	268.5	275.5	Reference	
Albumin alone	0.615 (0.538–0.691)	0.201	298.9	305.9		
LAR alone	0.719 (0.645–0.793)	0.170	265.0	272.1	LAR vs. lactate	0.205
Lactate+Albumin	0.720 (0.646–0.794)	0.170	266.4	276.9		
Lactate, mmol/L + D-dimer, mg/L + PaO2/FiO2, mmHg (final model)	0.902 (0.864–0.940)	0.114	189.1	203.2	Final model vs. LAR	<0.001

LAR was defined as lactate/albumin. Scaling LAR by 100 does not affect AUC.

Restricted cubic spline analyses showed no statistically significant evidence of nonlinearity for lactate, D-dimer, or PaO2/FiO2 (Supplementary Table S2; Supplementary Figures S4–S6). LASSO sensitivity analysis is provided in Supplementary Table S3 and Supplementary Figures S2–S3. At lambda.min, LASSO retained age, sex, hypertension, diabetes mellitus, WBC, HGB, lactate, albumin, INR, D-dimer, CRP, pH, and PaO2/FiO2; at lambda.1se, it retained age, lactate, D-dimer, pH, and PaO2/FiO2. Thus, LASSO consistently retained the three final-model predictors while also selecting additional variables, supporting but not uniquely confirming the primary model. In the full-pipeline bootstrap, PaO2/FiO2, D-dimer, and lactate were retained in 100.0%, 83.1%, and 62.2% of resamples, respectively (Supplementary Table S6). Decision curve analysis suggested that the final model provided higher net benefit than LAR alone and default strategies across clinically plausible threshold probabilities (Figure 5).

**Figure 5:**
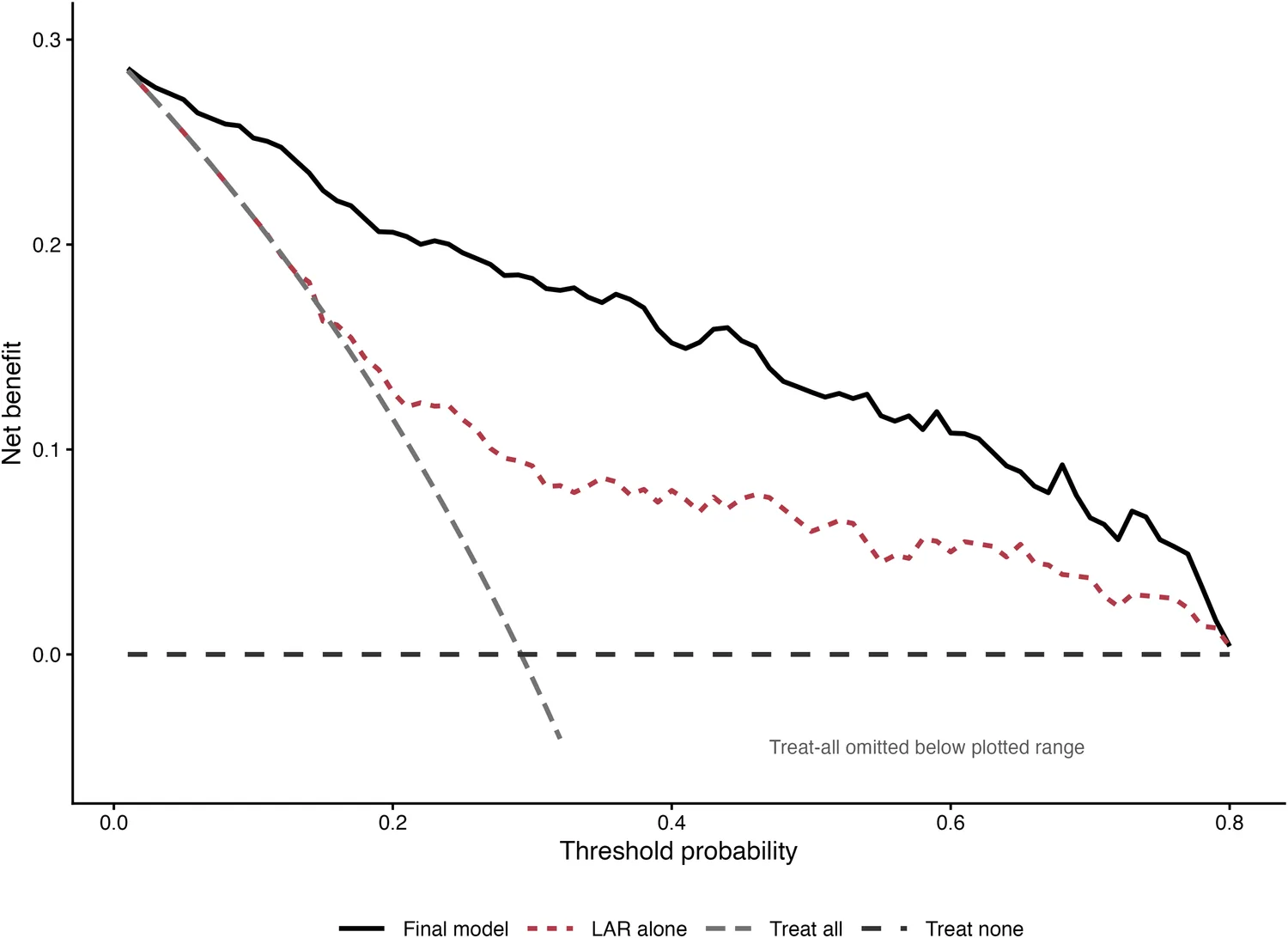
Decision curve analysis comparing the final selected model, LAR alone, treat-all, and treat-none strategies.

## Discussion

In this retrospective cohort of patients with septic shock, the final selected model retained three routinely available early variables: lactate, D-dimer, and PaO2/FiO2. The model combined markers of metabolic stress, coagulation/fibrinolysis activation, and oxygenation impairment, and showed good apparent discrimination with only modest optimism after 1,000 bootstrap resamples. The corrected AUC and calibration slope suggest that the signal was not driven solely by development-sample apparent performance, although the absence of external validation remains a central limitation. The model should therefore be interpreted as an internally validated risk-stratification tool rather than a clinical decision rule ready for implementation (23–26).

The selection of lactate is biologically and clinically consistent with shock pathophysiology. Elevated lactate in septic shock may reflect tissue hypoperfusion, impaired oxygen utilization, adrenergic stimulation, mitochondrial dysfunction, and reduced clearance; prior studies have consistently linked lactate level and lactate clearance to outcomes in severe sepsis and septic shock (9–11). In this study, lactate remained independently associated with 28-day mortality after adjustment for other variables that passed univariable screening. The cohort was identified from chart-documented septic shock during routine care, and lactate values were analyzed as early laboratory measurements within 24 h after clinical diagnosis of septic shock.

D-dimer contributed an additional coagulation-domain signal. Sepsis is tightly linked to endothelial injury, thrombin generation, fibrin deposition, fibrinolysis, and microvascular dysfunction, and these processes can progress toward sepsis-induced coagulopathy or disseminated intravascular coagulation (12–14). Prior work has shown that D-dimer may be associated with mortality in sepsis, although its performance as a single marker is often modest and context dependent (15). In the present model, D-dimer should not be viewed as an isolated diagnostic test. Its value is more plausibly incremental: it complements lactate by capturing a different biological pathway, namely coagulation and fibrinolytic activation, which may be particularly relevant in septic shock where microcirculatory derangement contributes to organ failure.

PaO2/FiO2 was retained as the oxygenation component of the model. Oxygenation impairment is clinically meaningful in sepsis because respiratory failure, pulmonary infection, acute lung injury, fluid loading, mechanical ventilation, and altered ventilation-perfusion matching may all influence mortality risk. PaO2/FiO2 is also embedded in ARDS classification and organ dysfunction scoring frameworks (6, 16). A large MIMIC-IV analysis reported that PaO2/FiO2 was independently associated with 28-day mortality in sepsis and highlighted the possibility of nonlinearity (17). In our restricted cubic spline analysis, however, there was no statistically significant evidence of nonlinearity for PaO2/FiO2 within this cohort. Therefore, the linear specification was retained for parsimony, interpretability, and consistency with the final logistic model.

The comparison with LAR is clinically informative. LAR has attracted attention because it combines lactate, a marker of metabolic stress, with albumin, a marker influenced by inflammation, capillary leak, hepatic synthesis, nutritional reserve, and fluid balance (18–22). In the present dataset, LAR alone did not significantly outperform lactate alone, whereas the final model showed substantially higher AUC and lower Brier score than LAR alone. This finding does not imply that LAR lacks prognostic value; rather, it suggests that a single metabolic-nutritional ratio may not capture the coagulation and oxygenation domains represented by D-dimer and PaO2/FiO2. The observed advantage of the final model should still be interpreted cautiously because it was evaluated in the same single-center cohort in which the model was developed.

A practical strength of the model is its reliance on variables that are commonly available early in patients with shock. The use of first available values within 24 h after clinical diagnosis of septic shock supports timely estimation and avoids requiring later trajectories before risk can be summarized. This simplicity is also a limitation. Static baseline values cannot represent the evolution of shock physiology, resuscitation response, lactate clearance, oxygenation trajectory, coagulation changes, vasopressor dose, renal replacement therapy, mechanical ventilation, source control, antimicrobial timing, or cumulative fluid balance. Dynamic models that incorporate serial lactate, D-dimer, oxygenation, platelet count, vasoactive support, and fluid balance may outperform a static model, but they require richer data collection and more careful temporal design.

Fluid management is particularly relevant to interpretation. Early crystalloid resuscitation, blood products, albumin administration, hemodilution, oxygen therapy, ventilator settings, and changes in FiO2 can alter measured lactate, albumin, D-dimer, and PaO2/FiO2. Oxygen strategy itself may influence physiologic interpretation in septic shock, as illustrated by trials evaluating hyperoxia and oxygen exposure in critical illness (28). Although initial resuscitation at our center was broadly consistent with contemporary sepsis guidance, detailed treatment-process variables were outside the prespecified laboratory-based predictor set. Future validation work should examine whether fluid balance, albumin administration, blood product transfusion, ventilator settings, and vasopressor dose modify model calibration or transportability across centers.

The lack of direct comparison with SOFA, SOFA-2, APACHE II, qSOFA, and NEWS2 is another important limitation. These scores incorporate clinical physiology, neurologic status, organ dysfunction, treatment intensity, and sometimes chronic health information beyond the laboratory and arterial blood gas variables evaluated in the present model (5–8, 29, 30). SOFA-2 has recently updated organ dysfunction assessment for contemporary ICU practice, including revised thresholds and organ-support variables, but the present retrospective dataset did not contain the complete elements required for reliable SOFA-2 reconstruction (29, 30). We therefore did not perform *post hoc* score comparisons based on incomplete score components. Future studies should prospectively collect the components of established and contemporary severity scores and compare discrimination, calibration, decision-curve net benefit, and clinical workflow utility against the present laboratory-based model.

Several methodological limitations also require emphasis. The study was retrospective and single-center, and the final analytic cohort included 250 patients and 73 deaths. Although the final refitted model included only three predictors, the broader development process evaluated 17 candidate variables and entered 12 variables into multivariable screening. Therefore, overfitting and selection instability remain possible despite bootstrap validation. The full-pipeline bootstrap produced more conservative optimism-corrected estimates than the fixed-final-model bootstrap, which supports cautious interpretation of model performance. We treated LASSO as a sensitivity analysis and used bootstrap resampling to quantify optimism, but neither approach substitutes for external validation. Complete-case cohort construction may introduce selection bias, particularly because 37 of 287 screened patients were excluded. Institutional treatment patterns, data availability, and case documentation may also affect model transportability.

Overall, the model is best framed as a transparent, parsimonious, early risk-estimation tool that may support risk stratification and research communication. It should not be presented as an artificial-intelligence model, a replacement for clinician judgment, or a tool that should guide treatment decisions by itself. Before routine use, the model requires external validation across hospitals, recalibration if baseline risk or predictor distributions differ, assessment with PROBAST-informed risk-of-bias principles, comparison against established clinical severity scores, and prospective evaluation of whether providing the risk estimate improves decisions or outcomes (23, 24).

## Conclusion

Using the original screening workflow, lactate, D-dimer, and PaO2/FiO2 were retained in the final model for 28-day mortality among patients with septic shock. The model showed good bootstrap-internally validated performance, but external validation is required before routine clinical use.

## Data Availability

The raw data supporting the conclusions of this article will be made available by the authors, without undue reservation.

## Supplementary material

SUPPLEMENTARY FIGURE S1 ROC comparison of LAR and candidate models.

SUPPLEMENTARY FIGURE S2 LASSO cross-validation curve.

SUPPLEMENTARY FIGURE S3 LASSO coefficient path.

SUPPLEMENTARY FIGURE S4 Restricted cubic spline analysis for lactate.

SUPPLEMENTARY FIGURE S5 Restricted cubic spline analysis for D-dimer.

SUPPLEMENTARY FIGURE S6 Restricted cubic spline analysis for PaO2/FiO2
